# The LUNG SAFE: a biased presentation of the prevalence of ARDS!

**DOI:** 10.1186/s13054-016-1273-x

**Published:** 2016-04-25

**Authors:** Jesús Villar, Marcus J. Schultz, Robert M. Kacmarek

**Affiliations:** CIBER de Enfermedades Respiratorias, Instituto de Salud Carlos III, Madrid, Spain; Multidisciplinary Organ Dysfunction Evaluation Research Network, Research Unit, Hospital Universitario Dr. Negrin, Barranco de la Ballena, s/n—4th floor, South Wing, Las Palmas de Gran Canaria, Spain; Department of Intensive Care, Academic Medical Center, Amsterdam, The Netherlands; Department of Respiratory Care, Massachusetts General Hospital, Boston, MA USA; Department of Anesthesiology, Harvard University, Boston, MA USA

## Abstract

The recent Large Observational Study to Understand the Global Impact of Severe Acute Respiratory Failure (LUNG SAFE) challenges current data on the prevalence of acute respiratory distress syndrome (ARDS). The LUNG SAFE investigators claimed that their data demonstrated the predictive validity of the Berlin criteria. Also, the LUNG SAFE showed a disturbingly large gap between scientific evidence and medical practice. All of these statements demand that we question the interpretations of the study’s findings.

*The fundamental feature of a scientific system is not that its propositions are verifiable, but that its propositions are falsifiable.*

Karl Popper

Acute respiratory distress syndrome (ARDS) is an acute and intense inflammatory response of the lungs that occurs as a result of either a direct or an indirect insult to the alveolar capillary membrane, causing increased permeability and subsequent interstitial and alveolar pulmonary edema. Characterized clinically by severe hypoxemia and bilateral radiographic infiltrates, ARDS usually occurs in previously healthy people. Usually, there is a latent period of 18–24 h between the insult and the development of the full-blown clinical syndrome. After this period, tachypnea, labored breathing, and cyanosis are observed. ARDS is generally confirmed by arterial hypoxemia and generalized infiltrates on chest radiograph, and the abnormalities in lung mechanics and oxygenation are better assessed once the patient is intubated and receiving mechanical ventilation (MV). Since 1967, little change in ventilator practice occurred until the publication of the pivotal ARMA trial [1] demonstrated that a lung-protective strategy using a tidal volume (V_T_) of 4–8 ml/kg of predicted body weight (PBW) and moderate levels of positive end-expiratory pressure (PEEP) improved survival. Since then, limitation of V_T_ to 6–8 ml/kg PBW and plateau pressure to a maximum of 30 cmH_2_O, and application of PEEP between 10 and 16 cmH_2_O represents the standard for MV in ARDS patients.

To date, efforts to diagnose or describe ARDS by one or more laboratory tests have failed. When defining ARDS, the specific ranges and conditions under which to evaluate the hypoxemia (most frequently assessed by the partial pressure of oxygen in arterial blood/fraction of inspired oxygen (PaO_2_/FiO_2_) ratio) have varied considerably. The original description [2], the American–European Consensus Committee [3], and the Berlin criteria [4] proved to be incapable of identifying uniform groups of patients in terms of severity and outcome [5–8] since none of them consider the sensitivity of PaO_2_/FiO_2_ to ventilator settings and the effects of routine care during the first 24 h for appropriate stratification, categorization, and prognostication [8]. There are no data that link a particular baseline PaO_2_/FiO_2_ to predictable structural changes in the alveolar capillary membrane. In addition, no biomarker has been described that is specific for ARDS, so it is plausible that ARDS prevalence is overestimated because many patients with acute hypoxemic respiratory failure from other diseases with bilateral pulmonary opacities and infiltrates [9] or patients with atelectasis, cardiogenic pulmonary edema, fluid overload, and obesity could be incorrectly diagnosed as having ARDS. Misdiagnosis can also occur if clinicians consider qualifying PaO_2_ values resulting from acute events unrelated to the disease process instead of considering only PaO_2_ values while patients are clinically stable [10].

The recent Large Observational Study to Understand the Global Impact of Severe Acute Respiratory Failure (LUNG SAFE) [11] challenges all of these statements and demands that we question the interpretations of its findings. The LUNG SAFE investigators reported an ARDS prevalence of 10.4 % of all ICU admissions and of 23.4 % of all patients receiving MV, a huge figure exceeding by an order of magnitude that expected from current clinical experience in Europe [12–15]. At least four sources of bias could explain this surprisingly epidemic figure.

First, 40 % of ARDS cases were included using an algorithm-recognition ARDS tool while participating clinicians did not diagnose them as ARDS. Considering all of the alternate causes of hypoxemia already listed that present as bilateral infiltrates on chest radiograph, it is quite challenging to disregard the clinician’s bedside interpretation that ARDS was not present for that of a computer algorithm which does not take into consideration these issues. How was the algorithm validated?

Second, more than 17 % of patients diagnosed with ARDS based on the Berlin criteria did not fulfill the criteria 24 h after routine care. Actual ARDS does not resolve in 24 h. Those patients who did not continue to meet criteria after 24 h most likely did not have ARDS and most likely had an alternate cause of hypoxemia and bilateral infiltrates that could be rapidly reversed [8–10].

Third, the study was performed in a short 4-week period during the winter of 2014, when prevalence of pulmonary infections, including H1N1 infection, had a seasonal peak [16] (pneumonia was reported to be almost 4-fold that of sepsis, a figure not supported by previous incidence studies) [12–15]. It is inappropriate to extrapolate data derived during a known worst seasonal period of a condition to represent the prevalence of the condition year around.

Finally, ICUs that did not enroll at least one ARDS patient during those 4 weeks were excluded from the analysis. This may be the most biasing problem of all. How can it be justified to eliminate data from groups originally designed to be part of the study of prevalence simply because they did not have a patient who met the criteria during the study period? The distribution of ARDS patients differs from institution to institution. Referral centers can be expected to have a higher prevalence than the average ICU, which may have periods without any ARDS patients. All should be considered in determining global prevalence.

Overall until now, the hospital mortality rate of patients with ARDS has remained >40 % in major observational studies [15]. Based on the *p* value for the 5 % absolute differences between the reported mortality rate of mild vs moderate ARDS and moderate vs severe ARDS, the LUNG SAFE investigators claimed that their data demonstrated the predictive validity of the Berlin criteria. What matters, however, is the probability that when you find that a result is “statistically significant” there is actually a real effect [17]. The Berlin definition does not help in segmenting patients into homogeneous subgroups with similar lung injury and outcome at its onset [8, 18]. Notably, there were no standard rules for measuring the PaO_2_/FiO_2_ at any time during the LUNG SAFE, and it was not reported how many patients within each category remained in the same category after the first 24 h of routine care. From this point of view, hospital mortality differences (calculated by us) between mild and moderate ARDS (*p* = 0.022) and between moderate and severe ARDS (*p* = 0.03) are meaningless since the use of nonstandardized PaO_2_/FiO_2_ measurement makes it difficult, if not impossible, to interpret the degrees of lung injury [18]. Of note, patients categorized as having severe ARDS, based on the Berlin definition, were younger and had fewer comorbidities and a worse outcome, a finding that contradicts previous knowledge [19]. There is still a need for a better ARDS definition—one that takes into consideration the patient’s actual ventilator settings and the fact that over the first 24 h of presumed ARDS, as the patient is stabilized, the true severity of the syndrome is identified and the status of many patients dramatically improves during this period.

Also, the LUNG SAFE investigators constructed 28-day survival curves for every ARDS category with missing patients in each category and assumed that patients discharged from the hospital before day 28 were alive. Mortality is a crucial outcome that should be measured very precisely. Causes of mortality were not reported. Patients with mild forms of ARDS do not die from ARDS but from the underlying disease (cancer, acquired immunodeficiency syndromes, stroke, advanced age), usually once discharged from the ICU [10]. Finally, surprisingly, the use of adjunctive therapies was analyzed after reclassifying patients using selectively the worst value of PaO_2_/FiO_2_ over the course of ICU stay instead of using the initial categorization, as the Berlin criteria mandate.

Besides all of these methodological sources of bias, a very relevant contribution of the LUNG SAFE is that it shows a disturbingly large gap between scientific evidence and medical practice. Most patients enrolled in this study were ventilated with V_T_ > 7 ml/kg PBW, PEEP < 10 cmH_2_O, and FiO_2_ > 0.6 and did not have their plateau pressure measured. A significant proportion of patients were ventilated with V_T_ > 9 ml/kg and less than 18 % of patients received PEEP > 11 cmH_2_O. It would be interesting to see whether there was a correlation between applied V_T_ and PEEP with worsening lung damage or with mortality. Why were proven therapies such as low-V_T_ MV, moderate to high levels of PEEP, and limitation of plateau pressure indeed ignored? Thus, it can only be assumed that there is still a huge need to assist the medical community in understanding the importance of lung-protective ventilation in all patients we mechanically ventilate.

## Ethics statement

Not applicable.

## Consent statement

Not applicable.

## Abbreviations

ARDS: acute respiratory distress syndrome
ARMA: A Respiratory Management in Acute Lung Injury/ARDS trial
LUNG SAFE: Large Observational Study to Understand the Global Impact of Severe Acute Respiratory Failure
MV: mechanical ventilation
PaO_2_/FiO_2_: partial pressure of oxygen in arterial blood/fraction of inspired oxygen
PBW: predicted body weight
PEEP: positive end-expiratory pressure
V_T_: tidal volume
